# The architecture of RNA polymerase fidelity

**DOI:** 10.1186/1741-7007-8-85

**Published:** 2010-06-22

**Authors:** Craig D Kaplan

**Affiliations:** 1 Department of Biochemistry and Biophysics, Texas A&M University, TAMU 2128, College Station, TX 77843-2128, USA

## Abstract

The basis for transcriptional fidelity by RNA polymerase is not understood, but the 'trigger loop', a conserved structural element that is rearranged in the presence of correct substrate nucleotides, is thought to be critical. A study just published in *BMC Biology *sheds new light on the ways in which the trigger loop may promote selection of correct nucleotide triphosphate substrates.

See research article http://www.biomedcentral.com/1741-7007/8/54

## 

Replicative or transcribing nucleic acid polymerases must produce complementary copies of nucleic acid templates by a mechanism that strikes a fine balance between fidelity and speed. For many of these enzymes, this is achieved by high selectivity for the correct substrate, with a proofreading step for removing incorrect nucleotides if the selection step fails (for a recent review see [1]). A number of options exist for proofreading by polymerases. DNA polymerases recognize noncomplementary base pairs and translocate them to a different domain or subunit of the enzyme for excision. For multisubunit RNA polymerases (RNAPs), detection and correction of nucleotide misincorporation both occur in the same active site: incorrect nucleotides may be released before they have been incorporated; or the misincorporation may cause the enzyme to pause and undergo an active site reorganization that, sometimes with the participation of extrinsic cofactors, favors a nucleolytic removal of RNA containing misincorporated substrates.

In broad terms, it is thought that binding of the correct complementary nucleotide to the DNA template in the RNAP active site induces closure of the site, with the correct alignment of critical amino acids for the polymerization reaction and thus efficient catalysis (see Figure 5 of [2]). The critical component in this structural rearrangement is the trigger loop, a flexible element of the largest subunit of RNA polymerase (the β' subunit in eubacterial RNAP, and the Rpb1 subunit in eukaryotic RNA polymerase II (Pol II)) that interacts with the substrate and other elements of the enzyme active site. Removal of the trigger loop causes a drastic reduction in both the speed and the accuracy of nucleotide addition [3], which is consistent with the general picture sketched above; and substitution mutants within the trigger loop can either increase or decrease the RNAP elongation rate *in vitro *in *Escherichia coli *or Pol II (see for example [4]), suggesting selection for an optimum balance of speed with accuracy. The exact role of the trigger loop in selective binding and catalysis has, however, remained unclear.

Recently, the understanding of the enzymatic activity of multisubunit RNAPs has reached a level of detail where the contributions of individual amino acid residues can be studied within an emerging structural framework, and this framework has provided the context for a kinetic analysis of mutant *Thermus aquaticus *RNAPs published in *BMC Biology *by Yuzenkova *et al. *[2], who now show how substrates can be screened at several steps in the synthetic process for their appropriateness before incorporation into a growing RNA chain, and make detailed suggestions on the structural basis for the discrimination. The screening mechanism at many of these steps consists in a reduction in catalytic efficiency that allows the enzyme to release mismatched substrates from the active site before incorporation can occur.

## The central role of the trigger loop

The evidence for the role of rearrangement of the active site comes from structural studies on highly structurally related RNAPs from many organisms [3], which have shown that the trigger loop can adopt multiple conformations, and studies on Pol II of *Sacharomyces cerevisiae *[5] and RNAP of *Thermus thermophilus *[6] in which it undergoes a structural reorganization that is dependent on the binding of a matched substrate. It has also been shown that the RNAP inhibitor Streptolydigin, which has effects similar to those of trigger loop deletion, restrains the trigger loop in a conformation in which it cannot interact with substrate [6,7].

One consequence of the structural reorganization that occurs on substrate binding is to place trigger loop residues proximal to substrate groups important for substrate recognition and phosphodiester bond formation, suggesting that the trigger loop may have a direct function in catalysis. One possibility is that a conserved histidine in the trigger loop (His1085 in Pol II, His936 in *E. coli *RNAP, and His1242 in *T. aquaticus *RNAP) might couple substrate recognition to catalysis by functioning as a general acid [5]. This would be consistent with the catalytic mechanism of several classes of single-subunit nucleic acid polymerases [8], in which a conserved basic residue involved in substrate recognition within a mobile domain also serves as a general acid, in this case for proton donation to the pyrophosphate leaving group. Further evidence for participation of the trigger loop in catalysis, but not in substrate binding, has come from biochemical studies on an *E. coli *trigger-loop deletion mutant that show no effect of the deletion on K_d_^app ^while *k*_cat _is strongly compromised for both nucleotide addition and the reverse reaction, pyrophosphorolysis [6].

The more recent studies of Zhang *et al. *[9] and Yuzenkova *et al. *[2] have focused on the effects of specific mutants of *E. coli *and *T. aquaticus *RNA polymerases, as well as trigger-loop deletion mutants, in an attempt to define the basis for selective nucleotide incorporation. Zhang *et al. *show that trigger loop mutant effects on NTP substrate incorporation during catalysis closely track mutant effects on pyrophosphorolysis, which is consistent again with a direct role for the trigger loop in catalysis through substrate-interacting residues [9]. However, the substitution of the uncharged amino acid alanine for the conserved histidine, or for Arg933 - or both - had only moderate effects on catalysis, arguing against a critical role for either of these basic residues as a general acid, as proposed in earlier studies [5,8].

## Multiple functions of the trigger loop in substrate selection

**Figure 1:**
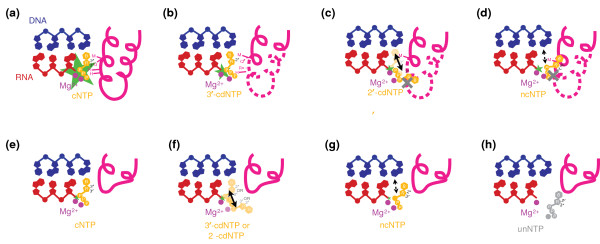
**The RNAP trigger loop makes multiple contributions to substrate selection**. The data from Yuzenkova *et al. *[2] are presented here as a generalized model for nucleotide triphosphate selectivity by multisubunit RNA polymerases, focusing on the trigger loop. A schematic elongation complex is shown with a nascent RNA (red), template DNA (blue), various NTP substrates (orange), catalytic Mg^2+ ^(magenta), and the trigger loop (pink). The green star illustrates the relative efficiency of catalysis with the specified substrate. **(a) **A catalytically favorable alignment or environment of a matched (cognate) NTP (cNTP) substrate relies on numerous trigger loop residues. Biochemical and structural evidence suggests that Met1238 positioning adjacent to the base moiety of the complementary NTP, Arg1239 and His1242 contacts with the triphosphate group, and Gln1235 interactions with the hydroxyls of the ribose moiety are important for rapid catalysis with matched substrates. Structural analyses indicate additional non-trigger loop contacts with triphosphates and ribose hydroxyls (not shown). **(b) **A 3'-cdNTP may utilize base-pairing with the template for positioning in the active site but is not added efficiently due to loss of particular trigger loop interactions, purportedly Gln1235 and its positioning of Arg1239. 3'-cdNTP substrates do not appear to compete with the trigger loop, consistent with localization of a 3'-cdNTP adjacent (base-paired) to the template in a Pol II crystal structure (K-M Larsson, personal communication). Even in a base-pairing conformation, catalysis is reduced. **(c) **2'-cdNTPs may also base-pair with the DNA template (occupancy in the addition site, or 'A site'); however, this occupancy is low at steady state in Pol II crystal structures, probably because of loss of a critical interaction with RNAP and the 2'-hydroxyl. Therefore, the trigger loop is unable to contribute efficiently to catalysis with 2'-cdNTPs. In addition, the 2'-cdNTPs may occupy a conformation at some frequency that is in conflict with a conformation of the intact trigger loop, leading to competition with the 2'-cdNTP and decreasing its affinity for the RNAP active site. This competition is sensitive to substitutions of Met1238, and is interpreted as indicating a requirement for Met1238 for trigger loop folding/movement. **(d) **Mismatched, non-cognate NTPs (ncNTPs) may not form complementary interactions with the template base, but the precise nature of ncNTP-template interactions will depend on the particular ncNTP-template mismatch. Because of these different orientations, catalysis with ncNTPs also varies by mismatch, but in all cases is reduced greatly by comparison with catalysis with cNTPs. Additionally, as with 2'-cdNTPs, an intact trigger loop may compete with ncNTPs, reducing the affinity of the enzyme for ncNTPs. This competition is also sensitive to substitutions in Met1238, again interpreted as indicating a requirement for Met1238 for trigger loop folding/movement. **(e) **In the absence of the trigger loop, NTPs are presumed still to base-pair effectively with the template DNA. However, they are not efficiently incorporated because of lack of critical trigger loop contacts. **(f) **2'-cdNTPs are not greatly affected by loss of the trigger loop, as the trigger loop does not strongly contribute to their incorporation because of inefficient positioning of the 2'-cdNTP for cooperation with the trigger loop. However, affinity for 2'-cdNTPs increases in the absence of the trigger loop because of lack of competition. **(g) **Certain ncNTPs may still be misincorporated in the absence of the trigger loop, without major loss of efficiency, because the trigger loop does not contribute greatly to their incorporation. Affinity for such ncNTPs increases, though, because of removal of trigger loop-ncNTP competition. **(h) **Other ncNTPs are discriminated against efficiently by the RNAP active site, depending on template base, indicating that their incorporation actually requires the trigger loop. In the absence of the trigger loop, they are considered unusable (unNTPs). The trigger loop still contributes to the overall selection for correct cNTPs over ncNTPs, by contributing more to catalysis of cNTPs than of ncNTPs even in the presence of trigger loop-independent discrimination between cNTPs and ncNTPs.

The new work now published in *BMC Biology *by Yuzenkova *et al. *[2] identifies some previously unrecognized mechanisms whereby RNAP discriminates different classes of nucleotide substrates. They conclude that the trigger loop is a kinetic selector for correct NTPs, functioning analogously to 'finger' domains of several classes of DNA polymerases by promoting catalysis of correct NTPs efficiently but incorrect substrates inefficiently, a notion that has already been proposed from a study of *S. cerevisiae *Pol II [10]. Their conclusions on the mechanism for discrimination of the distinct kinds of incorrect substrate are described in detail in Figure 1, and outlined below.

Substrate selection by RNA polymerase has two components, affinity of the polymerase for different substrates, and efficiency of catalysis by the enzyme for different substrates. Base-pairing of NTPs to the DNA template can provide differences in affinity between matched and mismatched substrates, but not between matched NTPs and matched dNTPs (which can base-pair as well). Other elements of a matched NTP substrate may be recognized, and structural studies give us an idea of how this may occur. Met1238 may be positioned directly adjacent to the base of an NTP base-paired with the template. Arg1239 and His1242 recognize the triphosphate moiety of the matched base in position for addition. Gln1235 appears to contribute to recognition of the 2'-OH or 3'-OH group on the ribose of the matched NTP. Much of this recognition is proposed to be subsequent to NTP binding and trigger loop rearrangement, and therefore part of an induced fit/kinetic selection of a matched NTP. The results of the studies of Zhang *et al. *are also consistent with this model: *E. coli *RNAP trigger loop residues Met932, Arg933, and His936, which are homologous to *T. aquaticus *Met1238, Arg1239 and His1242, contribute to catalysis of NTP substrates, not affinity [9]. However, Zhang *et al. *conclude from their *E. coli *experiments, contrary to proposals from *S. cerevisiae *Pol II work, and the conclusions of Yuzenkova *et al.*, that the *E. coli *trigger loop is not the major contributor to selection for matched, cognate NTPs (cNTPs) over non-cognate NTPs (ncNTPs) or 2'-cdNTPs: some of the results from the two studies are compared in Table 1, showing the difference in the magnitude of the contribution of the trigger loop in the *E. coli *and *T. aquaticus *studies.

**Table 1:** The contribution of the trigger loop compared in the *E. coli *and the *T. aquaticus *studies

***T. aquaticus *mutation **[2]	**Substrate/fold defect relative to WT (*k***_**pol**_**[cGTP])**	***E. coli *mutation **[9]	**Substrate/fold defect relative to WT (*k***_**CTP**_^**25 μM**^**)**
Arg1239Ala	GTP/~48	Arg933Ala	CTP/4
His1242Ala	GTP/~100	His936Ala	CTP/6
Arg1239Ala/His1242Ala	GTP/~1400	Arg933Ala/His936	CTP/24
		Ala	
Met1238Ala	GTP/~1800	Met932Ala	CTP/70
ΔTL	GTP/~62,500	TL^LTPP^*	CTP/12,000

***T. aquaticus *mutation**[2]	**Substrate/fold selectivity for cGTP**	***E. coli *mutation **[9]	**Substrate/fold selectivity for cATP**

WT *k*_pol_^app^	2'-cdATP/~1800	WT *k*_pol_^app^	ND
WT K_m_	2'-cdATP/~20	WT K_m_	ND
ΔTL *k*_pol_^app^	2'-cdATP/~1.2	ΔTL *k*_pol_^app^	2'-cdATP/~27
ΔTL K_m_	2'-cdATP/~1.4	ΔTL K_m_	2'-cdATP/~4

ND = Not determined. *k*_pol_^app ^= apparent *k*_pol_. *TL^LTPP ^is a double-proline substituted mutant in the *E. coli *TL proposed to compromised folding.

It seems clear that critical trigger loop residues in *T. aquaticus *provide the bulk of its function, while homologous residues in *E. coli *make a smaller contribution to trigger loop function. However, technical limitations did not allow Zhang *et al. *to calculate directly selectivity of the *E. coli *RNAP for a cNTP over a 2'-cdNTP, leaving open the question of the role of the trigger loop in this discrimination, whereas Yuzenkova *et al. *measured this directly and conclude that the trigger loop is critical for this process. Where both studies are once again in agreement is on the function of the basic residues in the trigger loop: in neither set of experiments do the effects of mutations to these residues support earlier proposals [5,8] that these function as a general acid.

## Unanswered questions

The differences between the *E. coli *RNAP and *T. aquaticus *RNAP suggest that caution should be exercised in drawing conclusions on RNAP mechanisms from a single system. These differences, as well as differences between *E. coli *RNAP and *S. cerevisiae *Pol II, may reflect adaptations resulting in similar but distinctive contributions of conserved residues in highly structurally homologous RNAPs. For example, all of these multi-subunit RNAPs function *in vivo *with accessory elongation factors that may alter RNAP activity, allowing differences in function or level of contribution of conserved residues to arise through evolution.

That said, there are some issues that require further careful experimental investigation. For example, Zhang *et al. *[9] and Yuzenkova *et al. *[2] report experimental results that are in direct conflict on the importance of the trigger loop in preventing misincorporation of GTP by similar or identical *E. coli *RNAP enzymes, and that thus cannot be explained as species differences. The next levels of experimentation will need to address the mechanism of RNAP translocation, approachable by biophysical means, and provide a deeper understanding of the catalytic mechanism. Proton inventory on the RNAP reaction in both wild-type and trigger-loop deletion mutants of RNAP, like those performed for other polymerase systems by the Cameron group [8], will be important for advancing our understanding of how multisubunit RNAPs may be distinct from other polymerases. Finally, molecular modeling, incorporating protein dynamics, at time scales that could capture both trigger-loop side chain fluctuations and trigger-loop folding or movement will be critical for a full accounting of the enzyme mechanism and the contributions of active site residues.

The results of Yuzenkova *et al. *underscore the several ways in which the trigger loop functions as a major determinant of RNAP substrate selection, and with other studies, suggests how the contributions of specific conserved trigger loop residues may differ in magnitude between RNAPs, perhaps reflecting functional diversification.
